# Supplementary material for: Digital health interventions for colorectal cancer screening uptake: A scoping review
Source: PLOS Digit Health. 2025 Sep 25;4(9):e0001028. doi: 10.1371/journal.pdig.0001028 (PMC12463253; doi:10.1371/journal.pdig.0001028)
Supplement: S1 Appendix — (DOCX) [file pdig.0001028.s001.docx]

**S1 Appendix**: PubMed Search Queries

#1: ("Colorectal Neoplasms"[MeSH Terms] OR "Colonic Neoplasms"[MeSH Terms] OR "Rectal Neoplasms"[MeSH Terms])

#2: ("digital intervention"[Text Word] OR "digital health intervention"[Text Word] OR "digital healthcare intervention"[Text Word] OR "mobile health"[Text Word] OR "mHealth"[Text Word] OR "electronic medical records"[Text Word] OR "patient portals"[Text Word] OR "decision aids"[Text Word] OR "telephone"[Text Word] OR "Internet"[Text Word] OR "Web-based"[Text Word] OR "telehealth"[Text Word] OR "telemedicine"[Text Word])

#3: ("Patient Education as Topic"[MeSH Terms] OR "patient education"[Text Word] OR "health literacy"[Text Word] OR "computer literacy"[Text Word] OR "digital literacy"[Text Word] OR "technology literacy"[Text Word])

#1 AND (#2 OR #3)

("Colorectal Neoplasms"[MeSH Terms] OR "Colonic Neoplasms"[MeSH Terms] OR "Rectal Neoplasms"[MeSH Terms]) AND ("Patient Compliance"[MeSH Terms] OR "Health Literacy"[MeSH Terms] OR "Patient Education as Topic"[MeSH Terms] OR "Consumer Health Information"[Text Word] OR "Uptake"[Text Word] OR ("Telemedicine"[MeSH Terms] OR "mHealth"[Text Word] OR "Mobile Health"[Text Word] OR "Patient Portals"[MeSH Terms] OR "Digital Health"[Text Word]))

#1 AND (#2 OR #3) Filters: Clinical Trial, Randomized Controlled Trial

("Colorectal Neoplasms"[MeSH Terms] OR "Colonic Neoplasms"[MeSH Terms] OR "Rectal Neoplasms"[MeSH Terms]) AND ("digital intervention"[Text Word] OR "digital health intervention"[Text Word] OR "digital healthcare intervention"[Text Word] OR "mobile health"[Text Word] OR "mHealth"[Text Word] OR "electronic medical records"[Text Word] OR "patient portals"[Text Word] OR "decision aids"[Text Word] OR "telephone"[Text Word] OR "Internet"[Text Word] OR "Web-based"[Text Word] OR "telehealth"[Text Word] OR "telemedicine"[Text Word] OR ("Patient Education as Topic"[MeSH Terms] OR "patient education"[Text Word] OR "health literacy"[Text Word] OR "computer literacy"[Text Word] OR "digital literacy"[Text Word] OR "technology literacy"[Text Word])) AND (clinicaltrial[Filter] OR englishabstract[Filter] OR randomizedcontrolledtrial[Filter])

**Note:** ClinicalTrials.gov searches were conducted to identify registered trials related to digital interventions for CRC screening. Matching publications were sought through PubMed and Google Scholar using study titles or NCT numbers. Backward citation tracking was performed in Web of Science for 46 of the 51 included studies, identifying 2,039 cited references (1,266 unique after deduplication). These records were screened using the same inclusion and exclusion criteria as the main database and register searches, resulting in two additional studies included in the final review.
