# Supplementary material for: Digital health interventions for colorectal cancer screening uptake: A scoping review
Source: PLOS Digit Health. 2025 Sep 25;4(9):e0001028. doi: 10.1371/journal.pdig.0001028 (PMC12463253; doi:10.1371/journal.pdig.0001028)
Supplement: S2 Appendix — Summarizes the PICOT (Population, Intervention, Comparator, Outcome, Time) framework analysis of 51 included studies. Presents structured tabular data of each study’s design, population, and key outcomes, along with full citations of all reviewed articles. This appendix supports the thematic synthesis presented in the Results section. (DOCX) [file pdig.0001028.s002.docx]

| **S2 Appendix.** PICOT Analysis of 51 Articles Reviewed | | | | | | |  |
| --- | --- | --- | --- | --- | --- | --- | --- |
|  |  |  |  |  |  |  |  |
| **First Author, Year** | **Population (P)** | **Intervention (I)** | **Comparator (C)** | **Outcome (O)** | **Time (T)** | **Site** |  |
| Basch, 2006 | Predominantly Black population in New York metropolitan area | Tailored telephone outreach | Direct mail approach | CRC screening uptake rate | 6 months | NYC urban minority, community health centers |  |
| Baus, 2020 | Residents of Appalachian region, WV | Data-informed practice facilitation via primary care systems | Not part of the initiative | Increase in baseline screening rate | 2014-2018 | WVU Health System, rural Appalachia |  |
| Champion, 2020 | Women aged 51-75 years nonadherent to breast and colon cancer screenings | Personally tailored messages via web or phone, or both | Usual care | Receipt of mammogram or stool test for CRC, dual screening adherence | 6 months | Indiana, Midwest primary care |  |
| Champion, 2023 | Rural women not up-to-date with breast, cervical, and CRC screenings | Tailored DVD with or without telephonic navigation | Usual care | Adherence to recommended cancer screenings, cost-effectiveness | 12 months | Indiana, multi-site clinics (urban/rural) |  |
| Chan, 2008 | Patients over 49 years with and without e-mail access | Internet-based personalized e-mail intervention (NetLET) | Control groups receiving printed reminders | Interest in and use of CRC screening | 2005-2005 | UT Houston Health Science Center |  |
| Cohen-Cline, 2014 | US population aged 50–81 years due for CRC screening | Interactive voice response (IVR) system to encourage CRC screening | Usual care | Adherence to CRC screening guidelines | 6 months | Kaiser Permanente Washington |  |
| Cooks, 2022 | Participants in a telehealth study | Racially similar and same gender matched virtual clinician | No race-matching | CRC screening intentions, perceptions of credibility and message relevance | N/S | Urban underserved Black communities, major metros |  |
| Coronado, 2023 | Participants aged 50–64 years, predominantly Hispanic/Latinx | Enhanced mailed FIT with advance notifications and automated calls | Standard mailed FIT with fewer reminders | 6-month FIT completion rate, overall CRC screening completion | 6 months | Southern California, AltaMed & Kaiser community clinics |  |
| Denizard-Thompson, 2020 | Vulnerable patients enrolled from community-based primary care practices | mPATH-CRC iPad decision aid | Usual care | Increase in CRC screening completion, mediated by various patient-level and system-level factors | 24 weeks | Wake Forest, 6 community practices, NC |  |
| Dodd, 2017 | Primary care patients aged 50 -74 years at average risk of CRC | Multicomponent primary care–based intervention including provision of an FOBT, printed information, and provider endorsement | Usual care without additional intervention | Completion of CRC screening 6 weeks post-recruitment | 6 weeks | NHS primary care, UK regional clinics |  |
| Elepaño, 2021 | Adults due for CRC screening | mHealth interventions promoting CRC screening | Usual care | CRC screening completion rates | N/S | Multi-country, pooled meta-analysis (NA, Europe, Asia) |  |
| Gautom, 2023 | Spanish-speaking Latino patients and staff in a FQHC | Virtual community engagement to develop colorectal cancer screening materials | N/S | Development and refinement of culturally relevant patient education materials for CRC screening | N/S | Texas FQHCs, Spanish-speaking Latino patients |  |
| Gomez, 2023 | Latinx community members aged 50-75 years in a church setting, not up-to-date with CRC screenings | Digital storytelling intervention in a church community | No intervention specified | CRC screening intention and perceptions post-intervention | N/S | San José State & Pentecostal Church, SF Bay Area |  |
| Goshgarian, 2022 | Average-risk managed care patients aged 50-75 years | Patient portal messaging before mailing FIT | Standard FIT mailing without portal messaging | CRC screening rates, time to screening | 6 months | UCLA Health System, Los Angeles |  |
| Green, 2013 | Adults aged 50-73 years not current for CRC screening | EHR-linked mailings with additive support: automated, telephone, nurse navigation | Usual care | Proportion of participants current for screening over 2 years | 2 years | Group Health Cooperative, 21 clinics, Seattle |  |
| Halm, 2023 | Patients aged 50–74 years from community-based clinics in a regional safety-net health system | Multiple system-level interventions: EHR reminders, quality measurement, mailed FIT | Prior interventions without mailed FIT | CRC screening rates over 10 years | 10 years | Parkland Health, Dallas County |  |
| Henderson, 2022 | High-risk cancer survivors | mHealth intervention with educational text-messages and resources for patients and PCPs | Control group with electronic resources only | CRC screening rates among high-risk cancer survivors | N/S | Parkland Health, Dallas County |  |
| Hirst, 2017 | Screening-eligible adults aged 60-74 years | Text-message reminder for fecal occult blood test | No reminder | CRC screening uptake via participation in English Bowel Cancer Screening Program | 18 weeks | England, 6 London CCGs (Croydon, Greenwich, etc.) |  |
| Hong, 2014 | Male subjects aged 50-59 years in Daegu, Korea | Combined telephone and postal interventions to promote screening | Control group with no intervention | Gastric and CRC screening uptake | 3 months | Daegu City Public Health Center, South Korea |  |
| Jerant, 2015 | Patients comprising three ethnicity/language strata | Tailoring knowledge acquisition for CRC screening | Non-tailored control | CRC screening knowledge and self-efficacy | N/S | USA, 5 cities, 26 primary care/FQHCs |  |
| Lafata, 2019 | Average-risk patients aged 50–75 years due for CRC screening | EHR-embedded patient portal program for post-visit CRC screening decision support | Usual care plus (with CRC screening information) | CRC screening uptake, patient-perceived benefits, barriers, and intent | N/S | Henry Ford Health System, Detroit region |  |
| Lohr, 2023 | Hispanic/Latino community | Digital storytelling intervention for breast, cervical, and CRC screening | N/S | Engagement and behavior change in screening | N/S | Mayo Clinic CBPR, Rochester, MN, Hispanic/Latino |  |
| López-Torres Hidalgo, 2016 | Persons over the age of 50 years | Interventions in primary care to increase CRC screening: written, telephone, group meeting | No briefing | Participation in CRC screening | 2 years | Albacete primary care, Spain (urban/rural) |  |
| Malo, 2021 | Vulnerable populations at CHCs in North Carolina | Centralized support for mailed FIT and patient navigation for follow-up colonoscopy | Usual care | CRC screening and follow-up rates | 6 months | North Carolina FQHCs, vulnerable populations |  |
| McIntosh, 2023 | Australian patients aged 49–60 years due for a National Bowel Cancer Screening Program (NBCSP) kit | SMS with or without motivational and instructional videos | Usual care | “CRC screening uptake via participation in National Bowel Cancer Screening Program | 6 months | Western Victoria, rural general practice, Australia |  |
| McIntosh, 2024 | Australian patients aged 50–60 years due for NBCSP kit | SMS including personalized message, motivational video, and instructional video | Usual care | Uptake of FIT screening | 12 months | Victoria regional general practice clusters, Australia |  |
| McQueen, 2019 | Unscreened adults aged 50–75 years | CRC screening information with narrative of a CRC survivor or screener | Information only | CRCS intentions and behaviors, emotional engagement | 1, 6, 12 months | Washington University, St. Louis, MO |  |
| Menon, 2022 | American Indian communities | Tailored navigator intervention for CRC screening | N/S | Screening completion rates | N/S | Arizona American Indian-serving clinics (tribal & urban FQHC) |  |
| Miller, 2018 | Vulnerable patients aged 50–75 years due for CRC screening | Digital health intervention (mPATH-CRC) via an iPad app | Usual care | Receipt of CRC screening, screening discussions, test orders | 24 weeks | NC Network Consortium, 6 primary care sites |  |
| Misra, 2011 | Adults aged 50–70 years, U.S., average-risk | Tailored interactive computer-based web intervention to promote CRC screening | Usual care, CDC web-based intervention (“Screen for Life”) | CRC screening uptake, cost-effectiveness | 12-month follow-up | UT Houston SPH & Kelsey-Seybold Clinic, Houston |  |
| Mitsutake, 2012 | Internet users in Japan | Association of eHealth literacy with knowledge and screening practice | Not applicable | CRC knowledge and screening practices | N/S | Japan national internet survey |  |
| Mosen, 2010 | Patients aged 51-80 at average risk for CRC | Automated telephone calls to promote FOBT screening | Usual care | Completion of FOBT | 6 months | Kaiser Permanente Northwest, Portland, OR |  |
| Muller, 2009 | Adults aged 50–75 years, U.S., HMO patients eligible for CRC screening | Secure email reminder system linked to EHR | Usual care, letter reminder group | CRC screening uptake rate | 3-month follow-up | Northwest Permanente HMO, Oregon |  |
| Muller, 2017 | Alaska Native and American Indian people aged 40-75 years | Text message reminders to increase CRC screening | Usual care | CRC screening rates | 6 months | Southcentral Foundation, Anchorage, Alaska |  |
| Rawl, 2024 | Low-income and minority patients who missed a colonoscopy appointment | Tailored DVD plus telephone-based patient navigation | Mailed DVD alone or usual care | Completion of CRC screening | 12 months | Indiana University & Ohio State Univ. Comprehensive Cancer Center |  |
| Richter, 2020 | Outpatients for colonoscopy | Digital pre-procedure instruction program | Usual care | Reduction in no-shows, cancellations, patient satisfaction with colonoscopy preparation | 3 months | MGH & Harvard Medical School, Boston, MA |  |
| Saini, 2023 | Veterans aged 70-75 years | Personalized multilevel intervention for appropriate use of CRC screening | Usual care | Screening uptake among older adults | N/S | US VA Ann Arbor Healthcare System |  |
| Schliemann, 2022 | Malaysians aged 50–75 years living in Segamat district | CRC screening intervention using home-testing and digital communication | N/S | CRC screening uptake, budgetary impact | N/S | (Missing, please clarify) |  |
| Scott, 2023 | Patients in a FQHC system aged 50–75 years at average CRC risk | Mailed stool test program with patient navigation | N/S | Screening completion rates, follow-up of positive FITs | 3 years | Central Texas FQHC, CommUnityCare, Dell Med School, UT Austin |  |
| Selva, 2019 | Patients eligible for their first CRC screening test in Catalonia aged 50-69 years | Brief phone intervention before standard screening invitation | Standard invitation alone | Participation in the CRC screening program | 6 months | Segamat District, Malaysia (SEACO & local health clinics) |  |
| Sequist, 2011 | Patients aged 50-75 years with an active health portal account and overdue for CRC screening | Personalized electronic patient messages via a health portal, promoting CRC screening | No electronic messages | Receipt of appropriate CRC screening | 4 months | Harvard Vanguard Medical Associates, Eastern MA |  |
| Stoop, 2012 | Adults aged 50-75 years eligible for CRC screening by colonoscopy | Pre-colonoscopy consultation via telephone | Face-to-face consultation | Response and participation rates in CRC screening | N/S | Erasmus & Academic Med Centre, Netherlands |  |
| Van Blarigan, 2020 | CRC survivors | Web-based dietary intervention with text messages | Wait-list control | Feasibility, acceptability, diet change, anthropometry, and biomarkers | 12 and 24 weeks | UCSF Helen Diller Cancer Center, SF |  |
| Vilaro, 2020 | Non-Hispanic Black women aged 50–73 in North Florida | Web-based, race- and gender-concordant VHA promoting guideline-concordant colorectal cancer screening (e.g., home FIT test) | N/S | Identified cues that improved perceived credibility, trustworthiness, and engagement with VHA-delivered messages | Iterative focus groups and think-aloud interviews conducted 2017–2018 | Univ. Florida STEM Translational Comm. Center, Gainesville |  |
| Vilaro, 2021 | Non-Hispanic Black women, mostly rural, ages 50–73 | Three evolving versions of a web-based VHA promoting CRC screening with a FIT test, refined using the Technology Acceptance Model framework | Iterative versions compared across time; no separate control group | Identified key changes that improved perceived social presence, trust, expertise, and ease of use | User-centered design cycles with focus groups/interviews, 2017–2018 | Univ. Florida, rural Southeast US |  |
| Vilaro, 2022 | Rural Black and White adults aged 50–73 in North Florida | Virtual Health Assistant (VHA)-delivered messages to promote colorectal cancer screening — perceptions explored via focus groups | N/S | Understanding differences in perceptions of trust, authority, and acceptability of a VHA to inform tailored communication strategies for CRC screening | Cross-sectional focus group design conducted between 2017–2018 | Univ. Florida STEM, rural North Florida community |  |
| Wilson-Howard, 2021 | Black men aged 50–73 | Development of a virtual clinician for CRC screening | N/S | Perceptions and credibility of the virtual clinician | Iterative design process over 18 months (2017–2018) | Bethune Cookman Univ., UF STEM, rural community sites |  |
| Wu, 2019 | Adults aged 50–74 in Guangzhou, China, who tested positive in preliminary CRC screening (high-risk factor questionnaire or iFOBT) but hadn’t yet undergone colonoscopy | SMS intervention to motivate colonoscopy completion | Control (no SMS intervention) | Colonoscopy adherence rates | 6-month follow-up with 1 month lag for outcome ascertainment | Guangzhou CRC screening, 12 districts, China |  |
| Wyse, 2023 | Patients with CRC undergoing surgery | Digital health intervention to support ERAS recommendations | Usual care | Hospital length of stay, emergency visits, quality of life, patient knowledge, health service utilization | Up to 90 days post-discharge, RCT runs 2022–2025 | Hunter New England Local Health District & John Hunter Hospital, NSW, Australia |  |
| Yen, 2021 | Primary care patients eligible for first-time CRC screening | Personalized colorectal cancer risk assessment | Generic CRC education script via phone (control arm) | Screening intent and completion, impact by risk score | 6 and 12 months | Stanford Univ. School of Medicine |  |
| Zalake, 2019 | Users interacting with internet-based virtual humans | VH health intervention to promote CRC screening | Other information mediums | User intentions to pursue health information, design efficacy | N/S | Univ. Florida STEM, rural North Florida & online US panels |  |

**A List of 51 Articles Analyzed in Full Citation**

- Basch CE, Wolf RL, Brouse CH, Shmukler C, Neugut A, DeCarlo LT, Shea S. Telephone outreach to increase colorectal cancer screening in an urban minority population. Am J Public Health. 2006;96(12):2246-2253. <https://doi.org/10.2105/ajph.2005.067223> PMID: 17077394
- Baus A, Wright LE, Kennedy-Rea S, Conn ME, Eason S, Boatman D, Gadde D. Leveraging electronic health records data for enhanced colorectal cancer screening efforts. J Appalachian Health. 2020;2(4):53. <https://doi.org/10.13023/jah.0204.07> PMID: 35702727
- Champion VL, Christy SM, Rakowski W, Lairson DR, Monahan PO, Gathirua-Mwangi WG, et al. An RCT to Increase Breast and Colorectal Cancer Screening. Am J Prev Med. 2020;59(2). <https://doi.org/10.1016/j.amepre.2020.03.008> PMID: 32690203
- Champion VL, Paskett ED, Stump TE, Biederman EB, Vachon E, Katz ML, Monahan PO. Comparative effectiveness of 2 interventions to increase breast, cervical, and colorectal cancer screening among women in the rural US: a randomized clinical trial. JAMA Netw Open. 2023;6. <https://doi.org/10.1001/jamanetworkopen.2023.11004> PMID: 37115541
- Chan EC, Vernon SW. Implementing an intervention to promote colon cancer screening through e-mail over the internet: lessons learned from a pilot study. Med Care. 2008;46(9). <https://doi.org/10.1097/MLR.0b013e3181805e3c> PMID: 18725823
- Cohen-Cline H, Wernli KJ, Bradford SC, Boles-Hall M, Grossman DC. Use of interactive voice response to improve colorectal cancer screening. Med Care. 2014;52(6):496-499. <https://doi.org/10.1097/MLR.0000000000000116> PMID: 24638119
- Cooks EJ, Duke KA, Neil JM, Vilaro MJ, Wilson-Howard D, Modave F, Krieger JL. Telehealth and racial disparities in colorectal cancer screening: A pilot study of how virtual clinician characteristics influence screening intentions. J Clin Transl Sci. 2022;6(1). <https://doi.org/10.1017/cts.2022.386> PMID: 35619640
- Coronado GD, Nyongesa DB, Escaron AL, Petrik AF, Thompson JH, Smith D, Leo MC. Effectiveness and Cost of an Enhanced Mailed Fecal Test Outreach Colorectal Cancer Screening Program: Findings from the PROMPT Stepped-Wedge Trial. Cancer Epidemiol Biomarkers Prev. 2023;32(11):1608-1616. <https://doi.org/10.1158/1055-9965.EPI-23-0597> PMID: 37566431
- Denizard-Thompson NM, Miller DP, Snavely AC, Spangler JG, Case LD, Weaver KE. Effect of a Digital Health Intervention on Decreasing Barriers and Increasing Facilitators for Colorectal Cancer Screening in Vulnerable Patients. Cancer Epidemiol Biomarkers Prev. 2020;29(8):1564-1569. <https://doi.org/10.1158/1055-9965.Epi-19-1199> PMID: 32381556
- Dodd N, Carey ML, Mansfield E, Oldmeadow C. Testing the effectiveness of a primary care intervention to improve uptake of colorectal cancer screening: A randomized controlled trial protocol. JMIR Res Protoc. 2017;6(5). <https://doi.org/10.2196/resprot.7432> PMID: 28490420
- Elepaño A, Fusingan AS, Yasay E, Sahagun JA. Mobile health interventions for improving colorectal cancer screening rates: A systematic review and meta-analysis. Asian Pac J Cancer Prev. 2021;22(10):3093-3099. <https://doi.org/10.31557/apjcp.2021.22.10.3093> PMID: 34710983
- Gautom P, Escaron AL, Garcia J, Thompson JH, Rivelli JS, Ruiz E, Coronado GD. Developing patient-refined colorectal cancer screening materials: application of a virtual community engagement approach. BMC Gastroenterol. 2023;23(1):179. <https://doi.org/10.1186/s12876-023-02774-8> PMID: 37221503
- Gomez V, Kandahari N, Curiel D, Carter A, Somkin CP, Allen AM. Digital storytelling as a tool to increase colorectal cancer screening intention in a Latinx church community. J Cancer Educ. 2023;38(6):1825-1833. <https://doi.org/10.1007/s13187-023-02338-0> PMID: 37434087
- Goshgarian G, Sorourdi C, May FP, Vangala S, Meshkat S, Roh L, Croymans DM. Effect of patient portal messaging before mailing fecal immunochemical test kit on colorectal cancer screening rates: a randomized clinical trial. JAMA Netw Open. 2022;5(2). <https://doi.org/10.1001/jamanetworkopen.2021.46863> PMID: 35119462
- Green BB, Wang CY, Anderson ML, Chubak J, Meenan RT, Vernon SW, Fuller S. An automated intervention with stepped increases in support to increase uptake of colorectal cancer screening: a randomized trial. Ann Intern Med. 2013;158(5):301-311. <https://doi.org/10.7326/0003-4819-158-5-201303050-00002> PMID: 23460053
- Halm EA, Nair RG, Hu E, Wang L, Lykken JM, Ortiz C, Skinner CS. Improving Colorectal Cancer Screening in a Regional Safety-Net Health System over a 10-Year Period: Lessons for Population Health. J Gen Intern Med. 2023;1-7. <https://doi.org/10.1007/s11606-023-08477-w> PMID: 37932541
- Henderson TO, Bardwell JK, Moskowitz CS, McDonald A, Vukadinovich C, Lam H, Kim K. Implementing a mHealth intervention to increase colorectal cancer screening among high-risk cancer survivors treated with radiotherapy in the Childhood Cancer Survivor Study (CCSS). BMC Health Serv Res. 2022;22. <https://doi.org/10.1186/s12913-022-08082-3> PMID: 35606736
- Hirst Y, Skrobanski H, Kerrison RS, Kobayashi LC, Counsell N, Djedovic N, et al. Text-message Reminders in Colorectal Cancer Screening (TRICCS): a randomised controlled trial. Br J Cancer. 2017;116(11):1408-1414. <https://doi.org/10.1038/bjc.2017.117> PMID: 28441381
- Hong NS, Kam S. Effectiveness of interventions to increase screening for gastric and colorectal cancer in Korea. Asian Pac J Cancer Prev. 2014;15(21):9147-9151. <https://doi.org/10.7314/apjcp.2014.15.21.9147> PMID: 25422193
- Jerant A, To P, Franks P. The effects of tailoring knowledge acquisition on colorectal cancer screening self-efficacy. J Health Commun. 2015;20(6):697-709. <https://doi.org/10.1080/10810730.2015.1018562> PMID: 25928315
- Lafata JE, Shin Y, Flocke SA, Hawley ST, Jones RM, Resnicow K, Tu SP. Randomised trial to evaluate the effectiveness and impact of offering postvisit decision support and assistance in obtaining physician-recommended colorectal cancer screening: the e-assist: Colon Health study—a protocol study. BMJ Open. 2019;9(1). <https://doi.org/10.1136/bmjopen-2018-023986> PMID: 30617102
- Lohr AM, Capetillo GP, Molina L, Goodson M, Smith K, Griffin JM, Sia IG. Development of a Digital Storytelling Intervention to Increase Breast, Cervical, and Colorectal Cancer Screening in the Hispanic/Latino Community: a Qualitative Evaluation. J Cancer Educ. 2023;1-8. <https://doi.org/10.1007/s13187-023-02389-3> PMID: 38044415
- López-Torres Hidalgo J, Rabanales Sotos J, Simarro Herráez MJ, López-Torres López J, Campos Rosa M, López Verdejo M. Effectiveness of three interventions to improve participation in colorectal cancer screening. Rev Esp Enferm Dig. 2016;108(6):315-322. <https://doi.org/10.17235/reed.2016.4048/2015> PMID: 27055722
- Malo TL, Correa SY, Moore AA, Ferrari RM, Leeman J, Brenner AT, Reuland DS. Centralized colorectal cancer screening outreach and patient navigation for vulnerable populations in North Carolina: study protocol for the SCORE randomized controlled trial. Implement Sci Commun. 2021;2:1-12. <https://doi.org/10.1186/s43058-021-00194-x> PMID: 34620250
- McIntosh JG, Emery JD, Wood A, Chondros P, Goodwin BC, Trevena J, Jenkins MA. SMARTERscreen protocol: a three-arm cluster randomised controlled trial of patient SMS messaging in general practice to increase participation in the Australian National Bowel Cancer Screening Program. Trials. 2023;24(1):723. <https://doi.org/10.1186/s13063-023-07756-5> PMID: 37957680
- McIntosh JG, Jenkins M, Wood A, Chondros P, Campbell T, Wenkart E, Emery JD. Increasing bowel cancer screening using SMS in general practice: the SMARTscreen cluster randomised trial. Br J Gen Pract. 2024;74(741). <https://doi.org/10.3399/BJGP.2023.0230> PMID: 38164588
- McQueen AMY, Caburnay C, Kreuter M, Sefko J. Improving adherence to colorectal cancer screening: a randomized intervention to compare screener vs. survivor narratives. J Health Commun. 2019;24(2):141-155. <https://doi.org/10.1080/10810730.2019.1587109> PMID: 30924402
- Menon U, Lance P, Szalacha LA, Candito D, Bobyock EP, Yellowhair M, Hatcher J. Adaptation of colorectal cancer screening tailored navigation content for American Indian communities and early results using the intervention. Implement Sci Commun. 2022;3(1):6. <https://doi.org/10.1186/s43058-022-00253-x> PMID: 35090575
- Miller DP Jr, Denizard-Thompson N, Weaver KE, Case LD, Troyer JL, Spangler JG, Lawler D, Pignone MP. Effect of a Digital Health Intervention on Receipt of Colorectal Cancer Screening in Vulnerable Patients: A Randomized Controlled Trial. Ann Intern Med. 2018;168(8):550-557. <https://doi.org/10.7326/m17-2315> PMID: 29532054
- Misra S, Lairson DR, Chan W, Chang YC, Bartholomew LK, Greisinger A, McQueen A, Vernon SW. Cost effectiveness of interventions to promote screening for colorectal cancer: a randomized trial. J Prev Med Public Health. 2011;44(3):101–110. https://doi.org/10.3961/jpmph.2011.44.3.101 PMID: 21617335
- Mitsutake S, Shibata A, Ishii K, Oka K. Association of eHealth literacy with colorectal cancer knowledge and screening practice among internet users in Japan. J Med Internet Res. 2012;14(6). <https://doi.org/10.2196/jmir.1927> PMID: 23149453
- Mosen DM, Feldstein AC, Perrin N, Rosales AG, Smith DH, Liles EG, Glasgow RE. Automated telephone calls improved completion of fecal occult blood testing. Med Care. 2010;48(7):604-610. <https://doi.org/10.1097/MLR.0b013e3181dbdce7> PMID: 20508529
- Muller D, Logan J, Dorr D, Mosen D. The effectiveness of a secure email reminder system for colorectal cancer screening. AMIA Annu Symp Proc. 2009;2009:457–461. PMID: 20351899
- Muller CJ, Robinson RF, Smith JJ, Jernigan MA, Hiratsuka V, Dillard DA, Buchwald D. Text message reminders increased colorectal cancer screening in a randomized trial with Alaska Native and American Indian people. Cancer. 2017;123(8):1382-1389. <https://doi.org/10.1002/cncr.30499> PMID: 28001304
- Rawl SM, Perkins SM, Tong Y, Katz ML, Carter-Bawa L, Imperiale TF, Champion V. Patient Navigation Plus Tailored Digital Video Disc Increases Colorectal Cancer Screening Among Low-Income and Minority Patients Who Did Not Attend a Scheduled Screening Colonoscopy: A Randomized Trial. Ann Behav Med. 2024. <https://doi.org/10.1093/abm/kaae013> PMID: 38470961
- Richter JM, Ha JB, Marx M, Campbell EJ, Pandolfi MC. A digital preprocedure instruction program for outpatient colonoscopy. Telemed e-Health. 2020;26(4):468-476. <https://doi.org/10.1089/tmj.2019.0050> PMID: 31298628
- Saini SD, Lewis CL, Kerr EA, Zikmund-Fisher BJ, Hawley ST, Forman JH, Vijan S. Personalized Multilevel Intervention for Improving Appropriate Use of Colorectal Cancer Screening in Older Adults: A Cluster Randomized Clinical Trial. JAMA Intern Med. 2023;183(12):1334-1342. <https://doi.org/10.1001/jamainternmed.2023.5656> PMID: 37902744
- Schliemann D, Ramanathan K, Tamin NSBI, O'Neill C, Cardwell CR, Ismail R, Donnelly M. Implementation of a colorectal cancer screening intervention in Malaysia (CRC-SIM) in the context of a pandemic: study protocol. BMJ Open. <https://doi.org/10.1136/bmjopen-2021-058420> PMID: 36581978
- Scott RE, Chang P, Kluz N, Baykal-Caglar E, Agrawal D, Pignone M. Equitable Implementation of Mailed Stool Test–Based Colorectal Cancer Screening and Patient Navigation in a Safety Net Health System. J Gen Intern Med. 2023;38(7):1631-1637. <https://doi.org/10.1007/s11606-022-07952-0> PMID: 36456842
- Selva A, Torà N, Pascual E, Espinàs JA, Baré M. Effectiveness of a brief phone intervention to increase participation in a population-based colorectal cancer screening programme: a randomized controlled trial. Colorectal Dis. 2019;21(10):1120-1129. <https://doi.org/10.1111/codi.14707> PMID: 31099455
- Sequist TD, Zaslavsky AM, Colditz GA, Ayanian JZ. Electronic patient messages to promote colorectal cancer screening: a randomized controlled trial. Arch Intern Med. 2011;171(7):636-641. <https://doi.org/10.1001/archinternmed.2010.467> PMID: 21149743
- Stoop EM, de Wijkerslooth TR, Bossuyt PM, Stoker J, Fockens P, Kuipers EJ, Dekker E, van Leerdam ME. Face-to-face vs telephone pre-colonoscopy consultation in colorectal cancer screening; a randomised trial. Br J Cancer. 2012;107(7):1051-1058. <https://doi.org/10.1038/bjc.2012.358> PMID: 22918392
- Van Blarigan EL, Kenfield SA, Chan JM, Van Loon K, Paciorek A, Zhang L, Venook AP. Feasibility and acceptability of a web-based dietary intervention with text messages for colorectal cancer: a randomized pilot trial. Cancer Epidemiol Biomarkers Prev. 2020;29(4):752-760. <https://doi.org/10.1158/1055-9965.EPI-19-0840> PMID: 31941707
- Vilaro MJ, Wilson‐Howard DS, Griffin LN, Tavassoli F, Zalake MS, Lok BC, Krieger JL. Tailoring virtual human‐delivered interventions: A digital intervention promoting colorectal cancer screening for Black women. Psycho‐Oncology. 2020;29(12):2048-2056. <https://doi.org/10.1002/pon.5538> PMID: 32893399
- Vilaro MJ, Wilson-Howard DS, Zalake MS, Tavassoli F, Lok BC, Modave FP, Krieger JL. Key changes to improve social presence of a virtual health assistant promoting colorectal cancer screening informed by a technology acceptance model. BMC Med Inform Decis Mak. 2021;21(1):196. <https://doi.org/10.1186/s12911-021-01549-z> PMID: 34158046
- Vilaro MJ, Wilson-Howard DS, Neil JM, Tavassoli F, Zalake MS, Lok BC, Krieger JL. A subjective culture approach to cancer prevention: rural black and white adults’ perceptions of using virtual health assistants to promote colorectal cancer screening. Health Commun. 2022;37(9):1123-1134. <https://doi.org/10.1080/10410236.2021.1910166> PMID: 33876658
- Wilson-Howard D, Vilaro MJ, Neil JM, Cooks EJ, Griffin LN, Ashley TT, Krieger JL. Development of a credible virtual clinician promoting colorectal cancer screening via telehealth apps for and by black men: qualitative study. JMIR Form Res. 2021;5(12). <https://doi.org/10.2196/28709> PMID: 34780346
- Wu Y, Liang Y, Zhou Q, Liu H, Lin G, Cai W, Gu J. Effectiveness of a short message service intervention to motivate people with positive results in preliminary colorectal cancer screening to undergo colonoscopy: a randomized controlled trial. Cancer. 2019;125. <https://doi.org/10.1002/cncr.32043> PMID: 30825395
- Wyse R, Smith S, Zucca A, Fakes K, Mansfield E, Johnston SA, Sanson-Fisher RW. Protocol: Effectiveness and cost-effectiveness of a digital health intervention to support patients with colorectal cancer prepare for and recover from surgery: study protocol of the Recover Esupport randomised controlled trial. BMJ Open. 2023;13(3). <https://doi.org/10.1136/bmjopen-2022-067150> PMID: 36878662
- Yen T, Qin F, Sundaram V, Asiimwe E, Storage T, Ladabaum U. Randomized controlled trial of personalized colorectal cancer risk assessment vs education to promote screening uptake. Am J Gastroenterol. 2021;116(2):391-400. <https://doi.org/10.14309/ajg.0000000000000963> PMID: 33009045
- Zalake M, Tavassoli F, Griffin L, Krieger J, Lok B. Internet-based tailored virtual human health intervention to promote colorectal cancer screening: design guidelines from two user studies. Proc ACM Int Conf Intell Virt Agents. 2019;19:73-80. PMID: 34027518

**Note:** Included studies are cited by author–year format for clarity. These studies were analyzed as part of the scoping review and are not listed in the manuscript’s reference section.
