# Supplementary material for: Digital health interventions for colorectal cancer screening uptake: A scoping review
Source: PLOS Digit Health. 2025 Sep 25;4(9):e0001028. doi: 10.1371/journal.pdig.0001028 (PMC12463253; doi:10.1371/journal.pdig.0001028)
Supplement: S1 Checklist — (DOCX) [file pdig.0001028.s003.docx]

**PRISMA-ScR Checklist**

Title: Digital Health Interventions for Colorectal Cancer Screening Uptake: A Scoping Review

| **Section and Topic** | **Item #** | **Checklist item** | **Location where item is reported** |
| --- | --- | --- | --- |
| **TITLE** |  |  |  |
| Identify the report as a scoping review | 1 | Identify the report as a scoping review in the title or abstract. | Title page, Abstract |
| **ABSTRACT** |  |  |  |
| Structured summary | 2 | Provide a structured summary that includes the objectives, eligibility criteria, participants, and the main findings. | Abstract |
| **INTRODUCTION** |  |  |  |
| Rationale | 3 | Describe the rationale for the scoping review in the context of what is already known. | Introduction |
| Objectives | 4 | State the objectives of the scoping review. | Introduction |
| **METHODS** |  |  |  |
| Eligibility criteria | 5 | Specify the eligibility criteria for the sources of evidence (e.g., participants, interventions, comparators). | Methods |
| Information sources | 6 | Specify all sources (e.g., databases, contact with authors) used to identify the evidence. Include the date of the last search. | Methods, Appendix A |
| Search | 7 | Present the full electronic search strategy for at least one database, including any limits used. | Methods, Appendix A |
| Selection of sources of evidence | 8 | State the process for selecting sources of evidence (e.g., number of reviewers, independent screening). | Methods |
| Data charting process | 9 | Describe the methods for charting data from sources of evidence (e.g., forms used, independently or in duplicate). | Methods, Appendix B |
| Data items | 10 | List and define all variables for which data were sought (e.g., participants, interventions, outcomes). | Methods, Appendix B |
| Critical appraisal of individual sources of evidence (optional) | 11 | If done, provide a rationale for conducting a critical appraisal of included sources. | Not applicable |
| Synthesis of results | 12 | Describe how the results were synthesized. | Results |
| **RESULTS** |  |  |  |
| Selection of sources of evidence | 13 | Give the number of sources of evidence screened, assessed for eligibility, and included in the review, with reasons for exclusions at each stage, ideally using a flow diagram. | Results, Figure 1 (PRISMA flowchart) |
| Characteristics of sources of evidence | 14 | Provide characteristics of the included sources of evidence (e.g., year of publication, country, intervention details). | Results, Appendix B |
| Critical appraisal within sources of evidence (optional) | 15 | If done, present data on the critical appraisal of included sources. | Not applicable |
| Results of individual sources of evidence | 16 | For each source of evidence, present the relevant data that were charted. | Appendix B, Results |
| Synthesis of results | 17 | Summarize and/or present the results as they relate to the review’s objectives and questions. | Results |
| **DISCUSSION** |  |  |  |
| Summary of evidence | 18 | Summarize the main results (e.g., which DHI interventions were effective). | Discussion |
| Limitations | 19 | Discuss limitations of the scoping review process (e.g., lack of critical appraisal, variability in data). | Discussion |
| Conclusions | 20 | Provide a general interpretation of the results and implications for future research, practice, or policy. | Discussion |
| **FUNDING** |  |  |  |
| Funding | 21 | Describe sources of funding for the review and other support. | Funding statement |
