# Supplementary material for: Digital health interventions for colorectal cancer screening uptake: A scoping review
Source: PLOS Digit Health. 2025 Sep 25;4(9):e0001028. doi: 10.1371/journal.pdig.0001028 (PMC12463253; doi:10.1371/journal.pdig.0001028)
Supplement: Attachment — Submitted filename: Response to Reviewers.docx [file pdig.0001028.s006.docx]

Division of Biomedical Informatics

Department of Internal Medicine

725 Rose Street, Room 230F,

Lexington, KY 40536-0003

(859) 218-0110 [sujinkim@uky.edu](mailto:sujinkim@uky.edu)

*5/7/2025*

Editor-in-Chief: Dr. Leo Anthony Celi

PLOS ONE DIGITAL HEALTH

[digitalhealth@plos.org](mailto:digitalhealth@plos.org)

***Response to Editor and Reviewers – Revised Manuscript Submission***

Manuscript Title: Digital Health Interventions for Colorectal Cancer Screening Uptake: A Scoping Review

Manuscript ID: PDIG-D-24-00417

Dear Editor and Reviewers,

Thank you for your thoughtful and constructive feedback. We have revised our manuscript in response to all eight points raised, with the aim of improving clarity, methodological rigor, and interpretive depth. All changes are highlighted in yellow in the revised manuscript, and we have also updated the PRISMA diagram, Table 2, and Appendix B accordingly.

Below is a point-by-point summary of our revisions, including where each change appears in the manuscript and which reviewer raised the issue:

**Point-by-Point Summary of Revisions**

1. **Explicit Research Questions in the Introduction**
   • *Reviewer #2 Concern:* The Introduction lacked clearly stated research questions.
   • *Revision:* We added three explicit research questions to the end of the Introduction.
   • *Location:* Page 3
2. **Justification of Health Literacy in Search Strategy**
   • *Reviewer #2 Concern:* Health literacy was included without conceptual justification.
   • *Revision:* We clarified the rationale for including “health literacy” as a key search term.
   • *Location:* Page 4 (Methods – Search Strategy)
3. **Clarify Inclusion/Exclusion Criteria**
   • *Reviewer #2 Concern:* Inconsistent inclusion of non-trial literature.
   • *Revision:* We revised the Eligibility Criteria to exclude protocols, systematic reviews, and meta-analyses unless embedded in trial design.
   • *Location:* Page 5 (Methods – Eligibility Criteria); PRISMA diagram (Figure 1); Appendix B updated accordingly
4. **Expand Explanation of Data Synthesis and Bibliometric Methods**
   • *Reviewers #2 & #3 Concern:* Thematic and bibliometric methods were underdescribed.
   • *Revision:* We added a full description of our synthesis approach and VOSviewer analysis.
   • *Location:* Page 6 (Methods – final paragraph before Results)
5. **Address Limited Outcomes Beyond Uptake Rates**
   • *Reviewer #1 Concern:* The review focused too narrowly on uptake outcomes.
   • *Revision:* We expanded the Discussion to include the need for long-term outcome data and comprehensive frameworks like RE-AIM.
   • *Location:* Page 14 (Discussion)
6. **Clarify ClinicalTrials.gov Usage**
   • *Reviewer #2 Concern:* The role of ClinicalTrials.gov as a source was unclear.
   • *Revision:* We clarified its use for identifying trial protocols and linking to published results.
   • *Location:* Page 4 (Methods – Search Strategy); Appendix A
7. **Replace Article IDs with Standard Citations**
   • *Reviewer #2 Concern:* Article IDs (e.g., Article 3) were non-standard.
   • *Revision:* We replaced all article IDs with author–year citations throughout the Results, Table 2, and Appendix B.
   • *Location:* Pages 7–13 (Results), Table 2, Appendix B
8. **Expand on Digital Literacy in Discussion**
   • *Reviewer #1 Concern:* DHL (digital health literacy) was insufficiently explored.
   • *Revision:* We expanded the Discussion to address DHL and its implications for tailoring digital interventions.
   • *Location:* Page 14 (Discussion)

**Additional Editorial Revisions**

- The **Financial Disclosure** section was revised to include author initials, grant numbers, and a statement of funder role: “The funders had no role in study design, data collection and analysis, decision to publish, or preparation of the manuscript.”
- The **Competing Interests** statement now reads: “The authors have declared that no competing interests exist.”
- **Funding source details and order** were made consistent between the manuscript and submission system.
- **Tables** were inserted as editable, cell-based objects within the main manuscript.
- **Figures** were uploaded separately in .TIF format per journal guidelines.
- We added a **“Supporting Information” legend section** after the References, clearly labeling S1 Appendix (Search Queries) and S2 Appendix (PICOT Analysis).

We are grateful for the opportunity to revise and resubmit this work to PLOS Digital Health. We hope these revisions fully address all concerns raised.

Sincerely,

Sujin Kim, PhD/Associate Professor	An Equal Opportunity University
