# Supplementary material for: Digital health interventions for colorectal cancer screening uptake: A scoping review
Source: PLOS Digit Health. 2025 Sep 25;4(9):e0001028. doi: 10.1371/journal.pdig.0001028 (PMC12463253; doi:10.1371/journal.pdig.0001028)
Supplement: Attachment — Submitted filename: Peer_Review_PLOS Digital Health.docx [file pdig.0001028.s005.docx]

The authors made significant and thoughtful revisions in response to the 1^st^ round peer review comments. These changes have greatly improved the manuscript. To further enhance the clarity and transparency, I would suggest the following:

1. The method section stated that “… we also conducted backward and forward citation tracking using Web of Science.” However, this step is not reflected in Figure 1. I recommend using the “PRISMA 2020 flow diagram for new systematic reviews which includes searches of databases, registers and other sources (available at [PRISMA 2020 flow diagram — PRISMA statement](https://www.prisma-statement.org/prisma-2020-flow-diagram)). This flow diagram template allows for the inclusion of backward/forward search under “Identification of studies via other methods.”
2. The methods section needs to clearly state that the data extraction and synthesis correspond to each research question (RQ). For example, what specific data were collected for RQ1, 2, and 3. Were data extraction from titles, abstracts, full texts, or metadata from Web of Science. How was the data analysis conducted for each RQ? Was RQ1 addressed by bibliometric keyword co-occurrence analysis only? Or combined with qualitative analysis?
3. In addition, more details are needed regarding the thematic analysis of co-occurring keywords. For example, what citation fields were used for keyword co-occurrence analysis? Author keywords? MeSH terms? Or Author keywords with MeSH? Or Web of Science assigned keywords/core concepts? Further, what is the threshold of keyword inclusion in VOSviewer? Only keywords appearing more than 3 times were included in the analysis and visualization? Did you use the default setting of VOSviewer for co-occurrence network mapping?

In the results, under RQ 1, the manuscript reads “Studies that integrated telephone consultations or used interactive voice response systems showed notable improvements in patient satisfaction and adherence to CRC screening rptocols…” Was this statement also from keyword co-ocurrence mapping?” If so, were these keywords (e.g., telephoine consulations,” “patient satisfaction” and “CRC screening” connected in Figure 2? What do the colors in Figure 2 represent? I would recommend adding legends to Figure 2 for color and size of the nodes.

4. The methods section also stated “… a bibliometric analysis using Web of Science was conducted to examine geographic trends and co-author networks.” There are no co-author networks reported in the results. Which RQ do the co-author networks address?
5. Results – Research Question 2: In Table 2: Classification of Digital Interventions by Delivery Mode, only 24 studies were listed and classified. The total number of included studies is 49. The authors need to explain why almost half of the studies were not classified in this table.
6. The authors stated that the included studies are heterogeneous in terms of sample size, being conducted in specific geographic or healthcare settings. In addition, “some studies were conducted outside the U.S., where different screening age recommendations may apply. It is worth extracting the study site information from the included 49 studies. The current bibliometric analysis refers only to the contributing authors/coauthors' countries, which may not reflect the actual study sites. Including this information would provide important context and strengthen the transparency of the findings and discussion.
