# Supplementary material for: Digital health interventions for colorectal cancer screening uptake: A scoping review
Source: PLOS Digit Health. 2025 Sep 25;4(9):e0001028. doi: 10.1371/journal.pdig.0001028 (PMC12463253; doi:10.1371/journal.pdig.0001028)
Supplement: Attachment — Submitted filename: Response to Reviewers 07162025.docx [file pdig.0001028.s007.docx]

Division of Biomedical Informatics

Department of Internal Medicine

725 Rose Street, Room 230F,

Lexington, KY 40536-0003

(859) 218-0110 [sujinkim@uky.edu](mailto:sujinkim@uky.edu)

*7/16/2025*

***Response to Editor and Reviewers – Revised Manuscript Submission***

Title: Digital Health Interventions for Colorectal Cancer Screening Uptake: A Scoping Review

Manuscript ID: PDIG-D-24-00417

Dear PLOSOne Digital Health editors and reviewers,

Thank you for your thoughtful and constructive feedback. We have carefully revised our manuscript in response to all points raised, with the goal of improving clarity, methodological rigor, and interpretive depth. All changes are highlighted in yellow in the revised manuscript. We have also updated the PRISMA 2020 flow diagram (Figure 1), the keyword co-occurrence network (Figure 2), Table 2, and Appendix B to address the specific concerns raised. Below is a point-by-point summary of our revisions, including where each change appears in the manuscript and which reviewer raised the issue:

**Point-by-Point Summary of Revisions**

Reviewer #1 – Comment #1
“Include younger and high-risk populations in future analyses.”

Response:
We thank the reviewer for highlighting the importance of addressing early-onset and high-risk populations in the context of evolving CRC epidemiology. In response, we have revised the *Discussion* to explicitly acknowledge the underrepresentation of younger populations in our findings, citing our PICOT analysis (*Table 1; Appendix B*) to support this point. We now emphasize the rising incidence of early-onset CRC (Siegel et al., 2020) and the USPSTF’s updated recommendation (Davidson et al., 2021) to reinforce the need for future research targeting younger and high-risk cohorts. (*See Discussion, pages 17–18, paragraph 1, 2.*)

Reviewer #1 – Comment #2
“Incorporate digital and health literacy assessments into study selection and analysis.”

Response:
We appreciate this thoughtful recommendation. In response, we have clarified in the *Methods* (page 7) that no formal digital health literacy (DHL) screening was applied during study selection, consistent with scoping review methodology. We have also expanded the *Discussion* (pages 18–19, paragraph 3) to note that only a minority of included studies reported DHL, and to highlight this as an important evidence gap. *Table 1* and our thematic synthesis emphasize the need for more rigorous DHL measurement in future research to better tailor digital interventions and promote equitable engagement.

Reviewer #1 – Comment #3
“Apply formal quality appraisal tools to assess study reliability.”

Response:
We appreciate this helpful suggestion. We have clarified in the *Methods* (page 7) that no formal risk of bias assessment or quality appraisal was conducted, in line with the scoping review framework which maps available evidence rather than evaluates effect sizes. We also note in the *Limitations* (Discussion, page 21, last paragraph) that the absence of formal quality appraisal may limit interpretation of individual study findings.

Reviewer #1 – Comment #4
“Encourage studies using innovative technologies like AI and app-based engagement.”

Response:
We thank the reviewer for this constructive recommendation. In response, we have added new text in the *Discussion* (page 20, discussion paragraph 7) highlighting the potential value of AI-driven risk stratification, decision support, and adaptive mHealth applications. We recommend that future research rigorously evaluate these approaches to enhance personalization, reach, and sustainability of CRC screening interventions.

Reviewer #1 – Comment #5
“Focus more on long-term outcomes and sustainability of DHIs.”

Response:
We thank the reviewer for this important point. We have expanded the *Discussion* (page 19 paragraph beginning “Fourth, our review predominantly shows…”) to emphasize the limited evidence on long-term effects and sustainability of digital health interventions for CRC screening. We now clearly recommend that future research incorporates longer follow-up periods, broader outcome measures, and assessments of sustained impact.

Reviewer #1 – Comment #6
“Enhance comparative analyses, particularly by CRC stage and intervention timing.”

Response:
We thank the reviewer for emphasizing this critical aspect. We have clarified in the *Discussion* (page 19, paragraphs beginning “Thirdly…” and “Lastly…”) that few included studies provide stage-specific or timing-specific comparisons, and we now highlight this as a critical gap. We recommend that future research design comparative analyses differentiating early versus late-stage CRC and explore how intervention timing affects patient engagement and screening uptake.

Reviewer #2 – Comment #1
“Revise Figure 1 to use the PRISMA 2020 flow diagram version that includes backward and forward citation tracking.”

Response:
We thank the reviewer for this valuable recommendation. In response, we have revised *Figure 1* (page 5) to align with PRISMA 2020 guidelines for systematic reviews that include searches of databases, registers, and other sources. We added a dedicated branch for “Citation searching (backward citation tracking)” under *Identification via other methods*. This was systematically conducted in Web of Science for 46 of the 49 included studies, yielding 2,039 cited references (1,266 unique after deduplication). These were screened using the same inclusion and exclusion criteria, resulting in two additional studies that met criteria, giving a final total of 51 included studies. We also updated the *Methods* (page 7), *Figure 2* legend (page 10), and supporting materials (*Tables 1 and 2; Appendix A – Search Queries; Appendix B – PICOT Analysis*) to ensure consistency.

Reviewer #2 – Comment #2
“The methods section needs to clearly state that the data extraction and synthesis correspond to each research question (RQ). For example, what specific data were collected for RQ1, 2, and 3. Were data extracted from titles, abstracts, full texts, or metadata from Web of Science? How was the data analysis conducted for each RQ?”

Response:
We thank the reviewer for this important point. We have revised the *Methods* (pages 7–8) to clarify how data extraction and synthesis were tailored to each research question. Specifically, we now state that data for RQ1 (trends and themes) were primarily extracted from PubMed titles and abstracts and analyzed through keyword co-occurrence mapping using VOSviewer, supported by thematic qualitative interpretation. Data for RQ2 (intervention characteristics) and RQ3 (comparative effectiveness and time factors) were extracted from full texts and structured using the PICOT framework. Metadata from Web of Science supplemented the bibliometric overview and backward citation tracking.

Reviewer #2 – Comment #3
“Add more details on the keyword co-occurrence analysis: fields used (Author keywords? MeSH? etc.), inclusion threshold, and whether VOSviewer defaults were used. Clarify whether statements in RQ1 come directly from the map and explain what the colors represent. Recommend adding a legend.”

Response:
We appreciate this detailed suggestion. We have revised the *Methods* (pages 7–8) to specify that the keyword co-occurrence map was generated using titles and abstracts from the final 51 PubMed records. A custom thesaurus was applied to merge synonyms, and VOSviewer’s full counting method with association strength normalization was used. A minimum occurrence threshold of six was set, resulting in 199 included terms. We verified that statements in RQ1 about telephone consultations, patient satisfaction, and CRC adherence are supported by mapped linkages. We added a clear legend to *Figure 2* (page 10) indicating that the colors represent the four synthesized thematic clusters: Patient-Centered Technology & Adherence, Behavioral Design & Personalization, Equity, Disparities & Community Engagement, and Clinical Workflow & Provider Interaction.

Reviewer #2 – Comment #4
“The methods section also stated ‘… a bibliometric analysis using Web of Science was conducted to examine geographic trends and co-author networks.’ There are no co-author networks reported in the results. Which RQ do the co-author networks address?”

Response:
We thank the reviewer for this helpful observation. We have clarified in the *Methods* (page 7) that the co-author network analysis was exploratory and linked to RQ1 to supplement the bibliometric mapping. Given its limited relevance to the main thematic synthesis, we removed explicit references to co-author network results from the *Results* to streamline focus on the primary findings.

Reviewer #2 – Comment #5
“In Table 2, only 24 studies were listed and classified. The total number of included studies is 49. The authors need to explain why almost half of the studies were not classified in this table.”

Response:
We thank the reviewer for this helpful comment. We have re-examined the entire dataset and updated *Table 2* (page 14) to classify all 51 included studies by digital delivery mode (Technology-only, Hybrid, or Human-facilitated). An explanatory note clarifies the classification method, and the *Results* section was revised to reflect this.

Reviewer #2 – Comment #6
“It is worth extracting the study site information... Including this information would provide important context and strengthen the transparency of the findings and discussion.”

Response:
We thank the reviewer for this insightful suggestion. We have systematically extracted and summarized the actual study sites for all 51 included studies. These details, including geographic location, healthcare setting, and target populations, are now provided in *Appendix B* and summarized in the *Results* and *Discussion*. This addition clarifies the real-world contexts in which the interventions were conducted and highlights site-specific differences, strengthening the transparency and interpretability of our findings.

We believe these revisions comprehensively address the reviewers’ comments and strengthen the transparency and methodological clarity of our scoping review.

Thank you for your consideration of our revised manuscript.

Sujin Kim, PhD/Associate Professor

An Equal Opportunity University
