# Supplementary material for: Digital health interventions for colorectal cancer screening uptake: A scoping review
Source: PLOS Digit Health. 2025 Sep 25;4(9):e0001028. doi: 10.1371/journal.pdig.0001028 (PMC12463253; doi:10.1371/journal.pdig.0001028)
Supplement: Attachment — Submitted filename: Response to Reviewers 08292025.docx [file pdig.0001028.s008.docx]

Division of Biomedical Informatics

Department of Internal Medicine

725 Rose Street, Room 230F,

Lexington, KY 40536-0003

(859) 218-0110 [sujinkim@uky.edu](mailto:sujinkim@uky.edu)

*8/29/2025*

***Response to Editor and Reviewers – Revision #3***

Manuscript Title: Digital Health Interventions for Colorectal Cancer Screening Uptake: A Scoping Review

Manuscript ID: PDIG-D-24-00417

Dear Reviewer and Editorial Team,

We sincerely thank you for your continued engagement and detailed comments that significantly enhanced the clarity and rigor of our work. We carefully reviewed all suggestions and revised the manuscript accordingly. Below is a detailed point-by-point response to Reviewer #2’s most recent comments, with references to specific page and line numbers from the updated clean manuscript.

Reviewer #2

Comment 1: Reduce repetitive writing, including repeated mention of PICOT and stats already in Figure 1.

Response:

We removed the redundant mention of PICOT in the Methods section (lines 137–147) and retained a concise definition upon first use (line 133). Figure 1’s title has been shortened to “PRISMA 2020 Flow Diagram of Study Selection (adapted from Page et al., 2021)” and no longer repeats search statistics shown in the figure itself.

(See: Methods, p. 7, lines 133–147; Figure 1, p. 8.)

Comment 2: Clarify definitions and significance of individual, contextual, and time-related factors mentioned in RQ2.

Response:

We added definitions for individual (e.g., age, gender), contextual (e.g., geography, access), technological (e.g., type of digital intervention), and time-related factors (e.g., timing/frequency of interventions) in the last paragraph of the Introduction.

(See: Introduction, p. 6, lines 112–119.)

Comment 3: Clarify what is meant by "conventional care" and illustrate with examples. Also reflect in Table 1.

Response:

We clarified in the Introduction and Results that conventional care typically included mailed reminders, in-clinic counseling, or routine outreach (see p. 6, lines 120–121; p. 20, lines 514–518).

Table 1 was revised to clearly define comparator practices and identify gaps in comparison across CRC stages and longitudinal impact.

(See: Introduction, p. 6, lines 120–121; Results, p. 20, lines 514–518; Table 1, p. 17.)

Comment 4: Clarify what “outcomes” means in the context of your PICOT analysis — do they include nonclinical metrics?

Response:

We revised the Methods to define “outcomes” as both clinical (e.g., screening uptake, adherence) and nonclinical (e.g., self-efficacy, knowledge, satisfaction). This clarification is also reflected in a note below Table 1.

(See: Methods, p. 8, lines 163–165; Table 1 Note, p. 17.)

Comment 5: Align aim with scoping review methodology by avoiding language suggesting evaluation of “effectiveness.”

Response:

We revised the aim on page 6 to emphasize “mapping current evidence” rather than assessing effectiveness.

(See: Introduction, p. 6, lines 108–110.)

Comment 6: Cite literature validating the use of VOSviewer. Avoid causal claims based on co-occurrence maps.

Response:

We now cite Van Eck & Waltman (2014) and Zupic & Čater (2015) to support use of VOSviewer. Language in the Results and Figure 2 has been revised to reflect observed research activity patterns, not causal inference.

(See: Methods, p. 9, line 175; Results, p. 13, lines 327–329.)

Comment 7: Qualify claim about underrepresentation of populations under 50. Only some studies report age.

Response:

We revised the Discussion to clarify that “among studies that reported age data,” few focused on adults under 50, limiting ability to assess representation of early-onset CRC populations.

(See: Discussion, p. 25, lines 613–616; Table 1, p. 17.)

We hope these revisions adequately address all remaining concerns and further strengthen the manuscript. Thank you again for your time, expertise, and helpful guidance.

Sincerely

Sujin Kim, PhD/Associate Professor

An Equal Opportunity University
